# Supplementary material for: Retrotransposon insertions can initiate colorectal cancer and are associated with poor survival
Source: Nat Commun. 2019 Sep 6;10:4022. doi: 10.1038/s41467-019-11770-0 (PMC6731219; doi:10.1038/s41467-019-11770-0)
Supplement: Supplementary file 1 — Supplementary Information [file 41467_2019_11770_MOESM1_ESM.pdf]

Supplementary Information

Retrotransposon insertions can initiate colorectal cancer and are associated with poor survival

Cajuso *et al*.

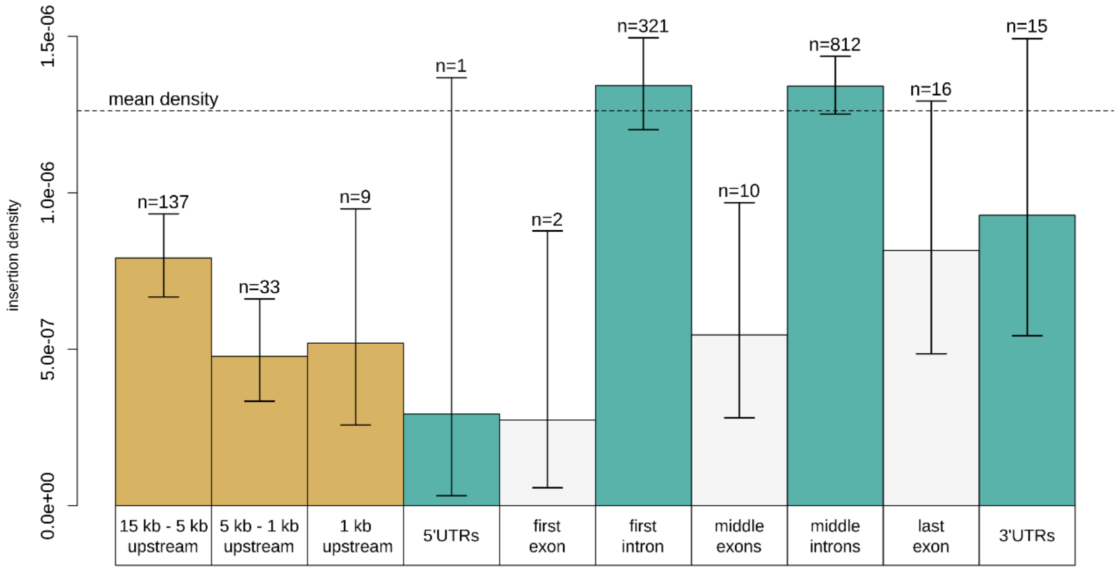

Supplementary Figure 1. Density plot of insertions in different genic regions. Annotation was extracted from the longest protein-coding transcript (n=20,167) and the longest 5' and 3' UTR were used. Last introns were included as part of middle introns. Binomial proportion 95% confidence intervals (Jeffreys intervals) are represented for each region. Mean density of the union of all the annotated regions is represented as a dashed line for reference.

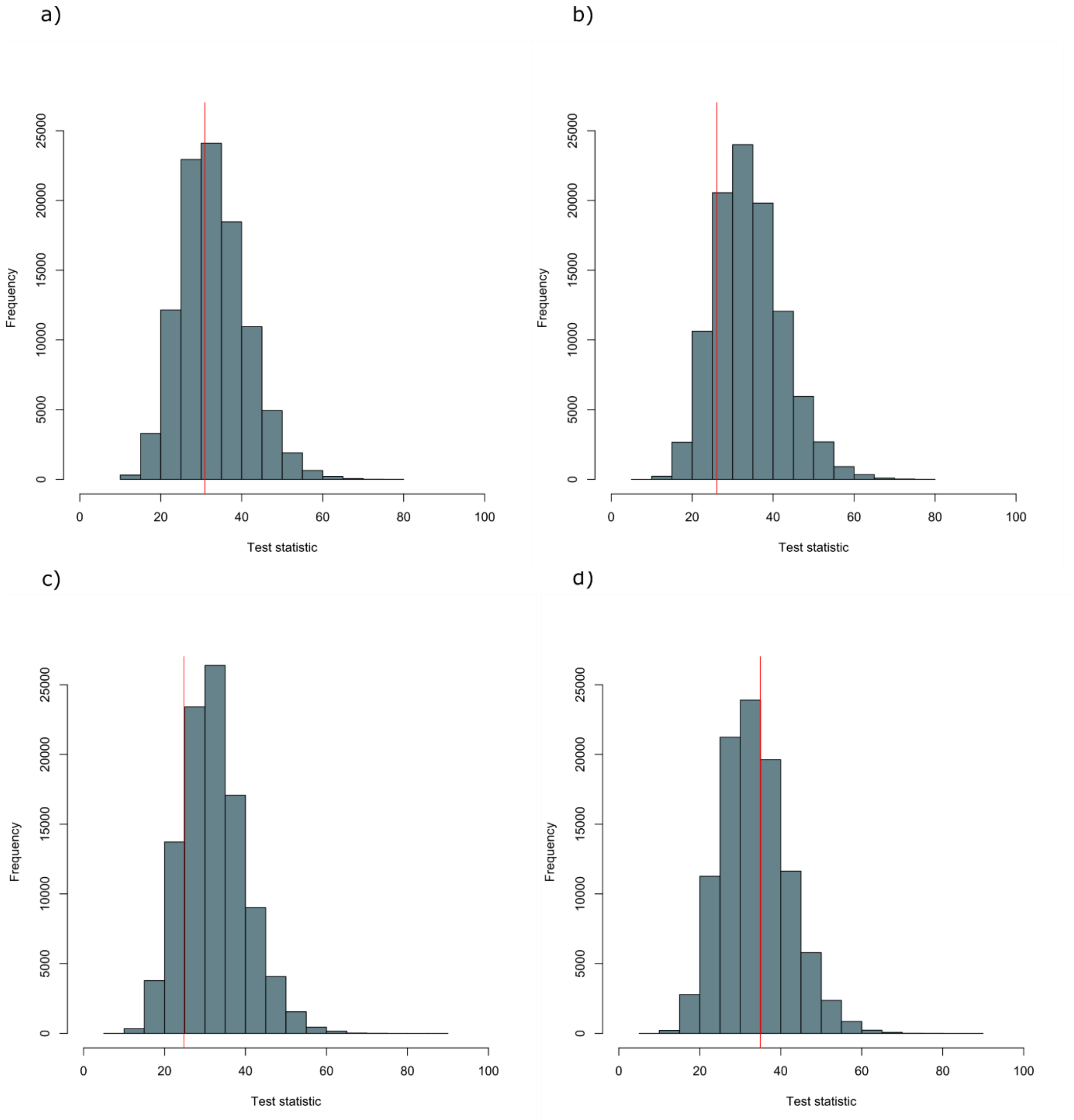

Supplementary Figure 2. Histograms of the sum-of-squared error statistic (SSE) (Chi-square test) from 100, 000 permutations. Each SSE statistic was calculated between permuted and uniform distribution of expression ranks of genes with insertion in close proximity. Red line refers to the observed SSE. **a)** Closest genes upstream an insertion loci, max distance 1 Mbp (n=453 insertions). **b)** Closest genes downstream an insertion loci, max distance 1 Mbp (n=435 insertions). **c)** Genes with an insertion (n=180 insertions). **d)** Closest gene (upstream or downstream) without any distance limitation (n=827 insertions).

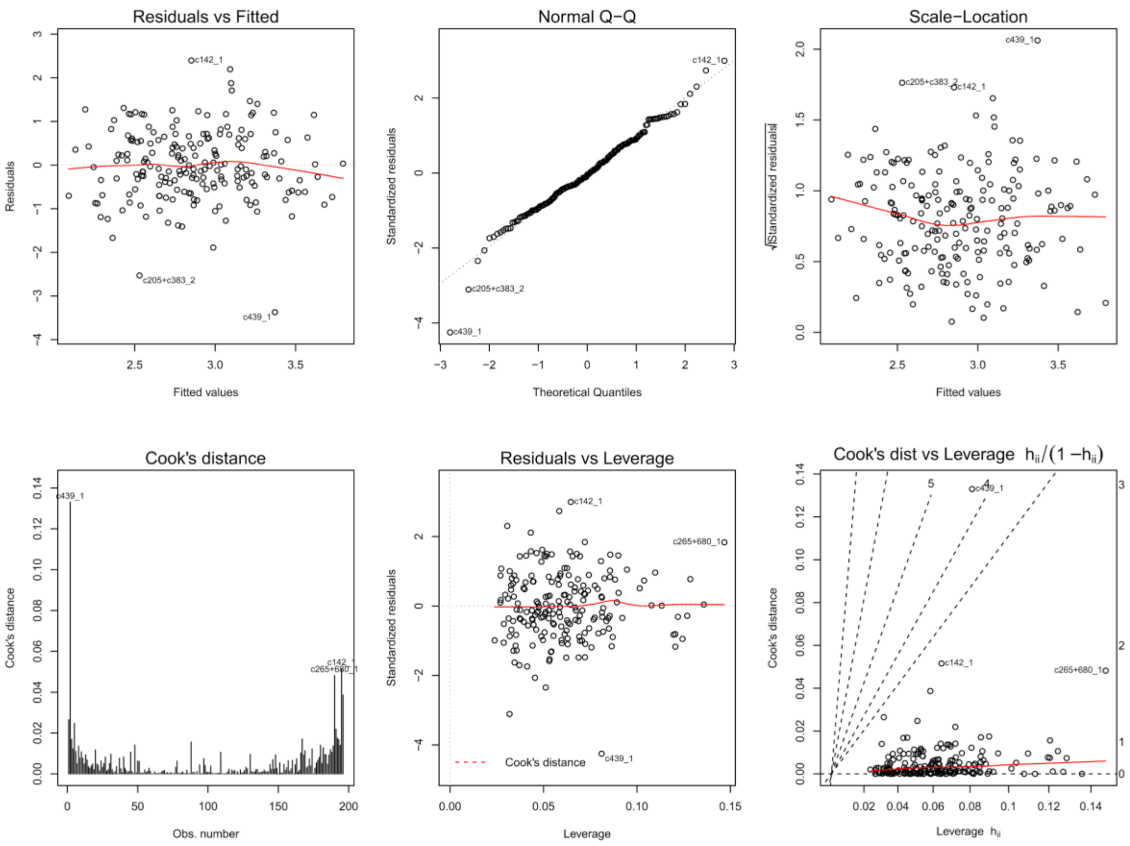

Supplementary Figure 3. Residuals plots against fitted values, theoretical quantiles and leverage. The associations with CIMP and the genomic fraction in allelic imbalance remained significant after removing a potentially influential observation from the multiple linear regression model (c439_1; p= 0.000224 and p=0.000460, respectively).

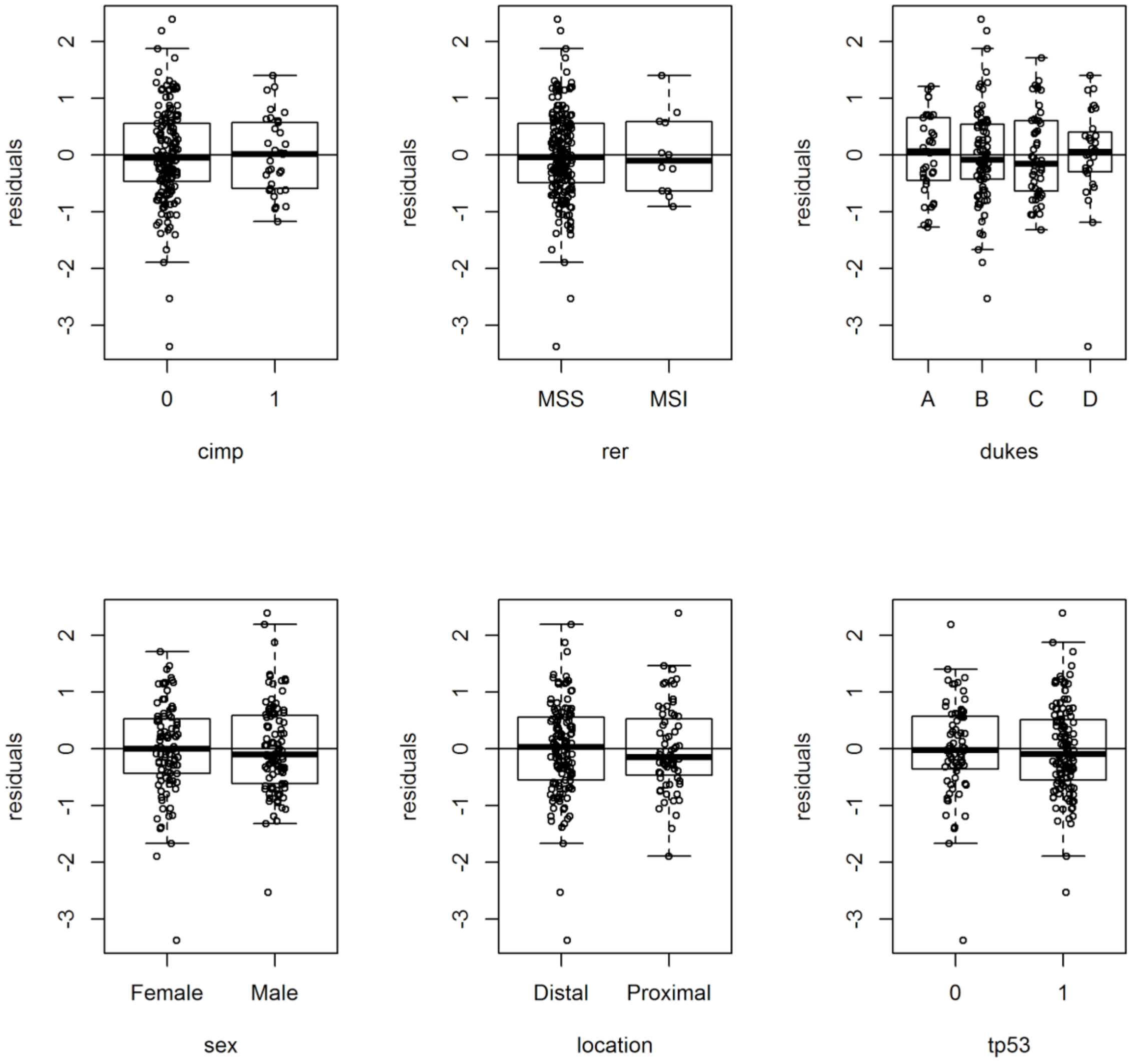

Supplementary Figure 4. Multiple linear regression model residuals for each categorical variable.

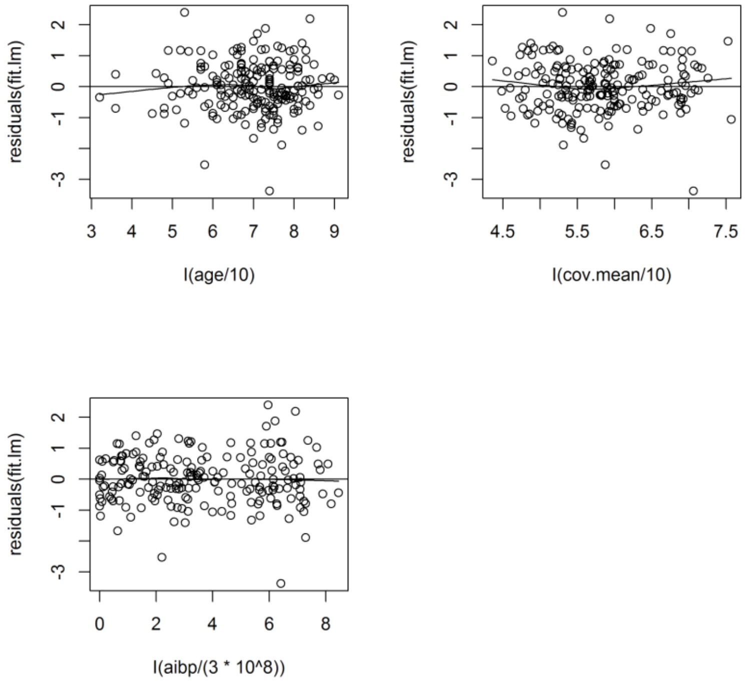

Supplementary Figure 5. Multiple linear regression model residuals for each continuous variable**.**

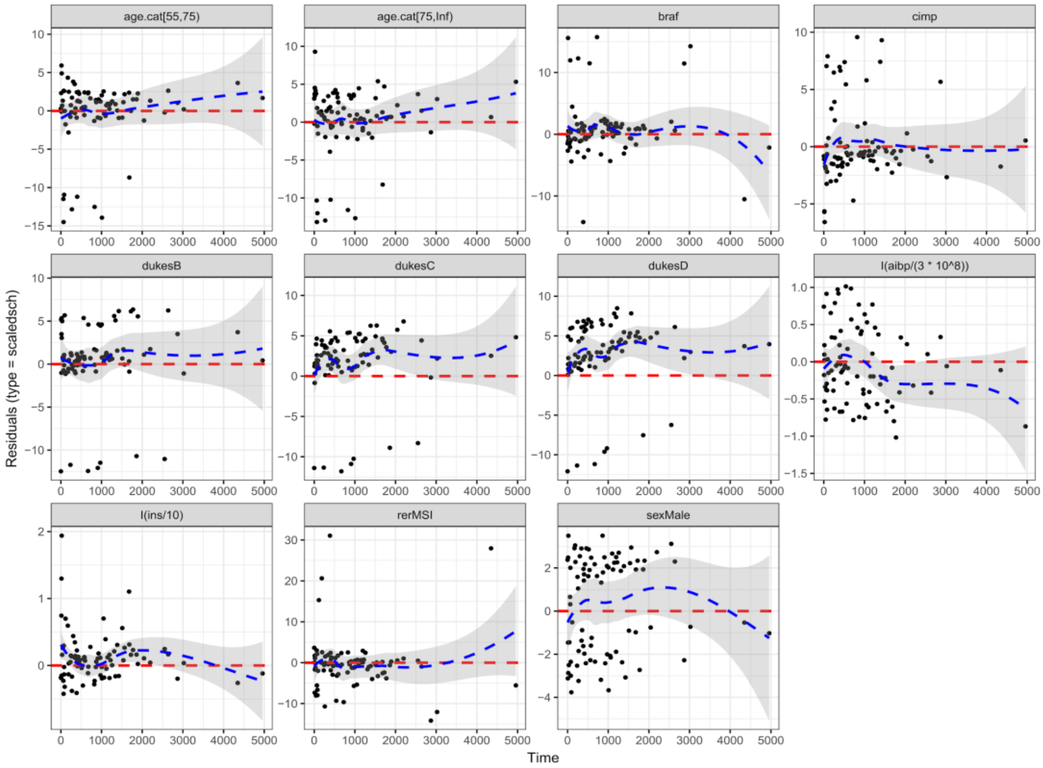

Supplementary Figure 6. Scaled Schoenfeld residuals on transformed time for each variable to assess for proportional hazards in the Cox proportional hazards model.

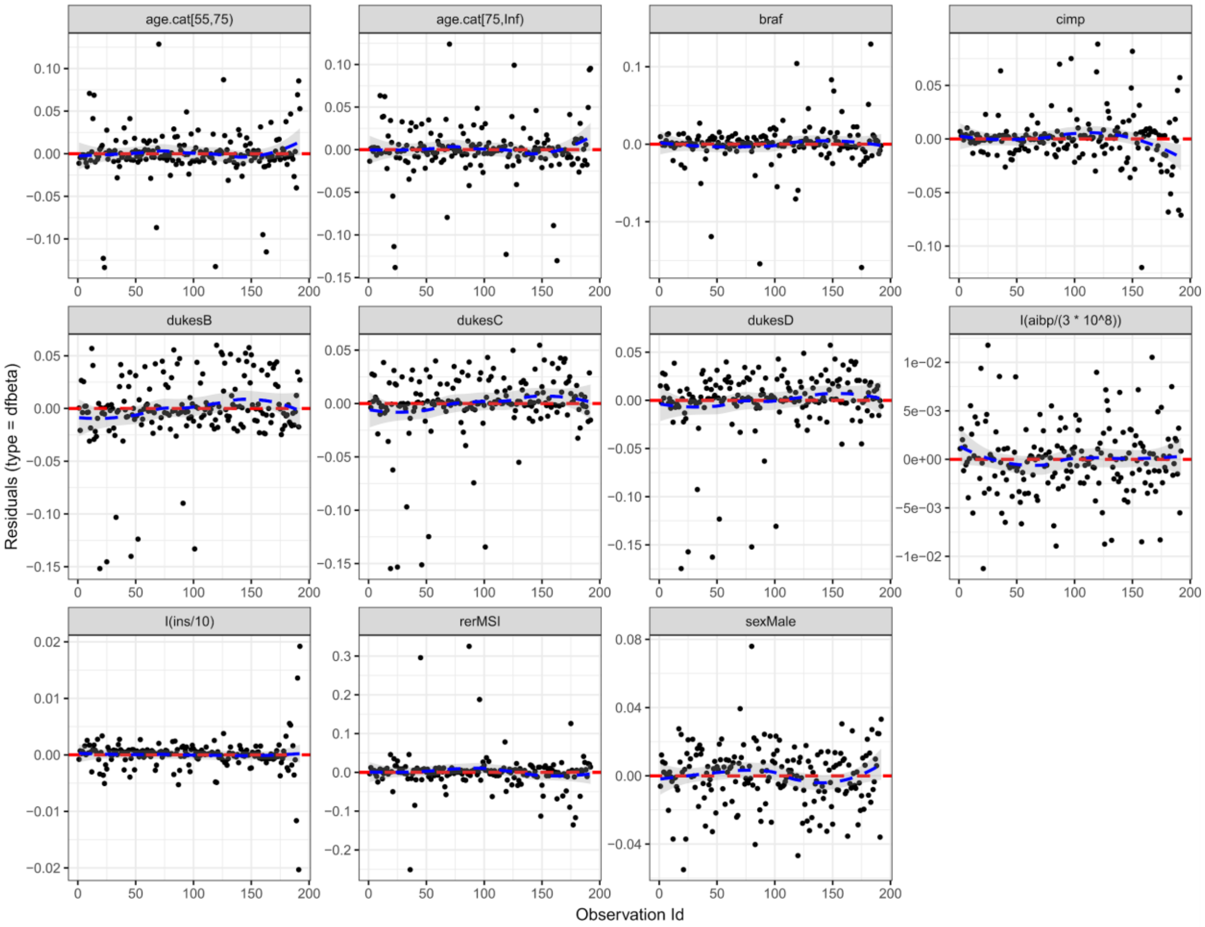

Supplementary Figure 7. Dfbeta residuals to assess for influential observations in the cox proportional hazards model.

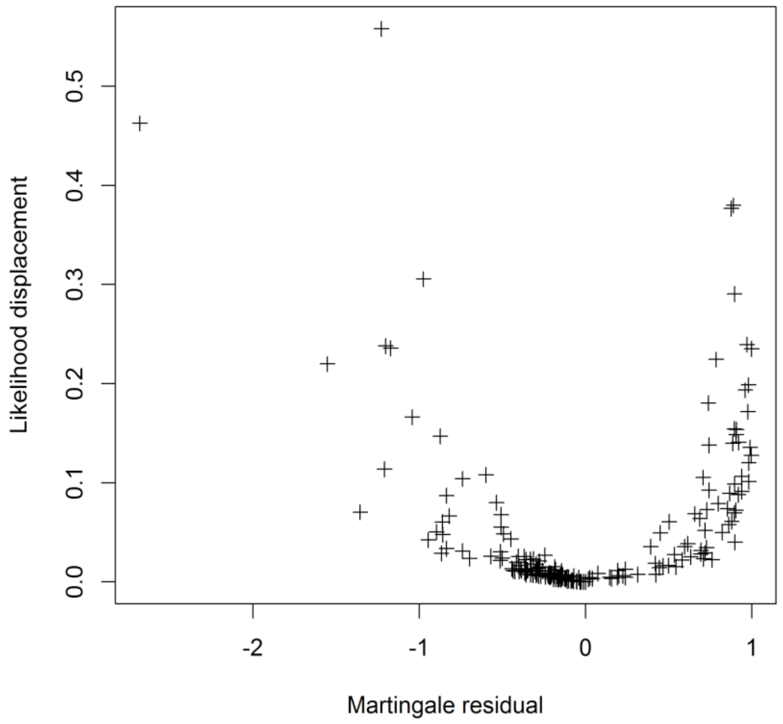

Supplementary Figure 8. Martingale residuals against likelihood displacement to assess for influential observations in the cox proportional hazards model.
