# Supplementary material for: Retrotransposon insertions can initiate colorectal cancer and are associated with poor survival
Source: Nat Commun. 2019 Sep 6;10:4022. doi: 10.1038/s41467-019-11770-0 (PMC6731219; doi:10.1038/s41467-019-11770-0)
Supplement: Supplementary file 3 — Description of Additional Supplementary Files [file 41467_2019_11770_MOESM3_ESM.pdf]

Description of Additional Supplementary Files

File Name: Supplementary Data 1
Description: Annotation of 5,072 somatic insertions detected in 202 colorectal tumor whole genomes. Exon hit, intron hit: insertion breakpoint in an exon or an intron in any transcript (GRCh37_87). "-", No data. c232.1T: colorectal adenoma. c779.1T, c465.1T and c927.1T: ultra-mutated tumors.

File Name: Supplementary Data 2
Description: List of protein-coding genes with at least one insertion. Number of insertions per gene, median TPM from 34 tumors, Cancer Gene census annotation and fragile site probability are included for each gene.

File Name: Supplementary Data 3
Description: List of protein-coding genes in fragile sites. Insertion count and fraction, median TPM, number of AI events and fraction, and the classification based on the ratio of insertions /AI used in **Figure 5** are included.

File Name: Supplementary Data 4
Description: List of L1 elements with at least one transduction. Cytogenetic band were converted with (https://www.ncbi.nlm.nih.gov/genome/tools/cyto_convert/).

File Name: Supplementary Data 5
Description: Clinical and molecular characteristics used in the multiple linear regression model and the cox proportional hazards model. CIMP: 0 = CIMP-low or CIMP:1= CIMP-high; *BRAF*, *KRAS*, *TP53*: 0 = wild type, 1 = mutation positive.
