# Supplementary material for: Retrotransposon insertions can initiate colorectal cancer and are associated with poor survival
Source: Nat Commun. 2019 Sep 6;10:4022. doi: 10.1038/s41467-019-11770-0 (PMC6731219; doi:10.1038/s41467-019-11770-0)
Supplement: Supplementary file 9 — Reporting Summary [file 41467_2019_11770_MOESM9_ESM.pdf]

nature research

Corresponding author(s): Lauri A. Aaltonen

Last updated by author(s): Jun 14, 2019

Reporting Summary

Nature Research wishes to improve the reproducibility of the work that we publish. This form provides structure for consistency and transparency in reporting. For further information on Nature Research policies, see Authors & Referees and the Editorial Policy Checklist.

Statistics

For all statistical analyses, confirm that the following items are present in the figure legend, table legend, main text, or Methods section.

n/a	Confirmed	
☐	☒	The exact sample size (*n*) for each experimental group/condition, given as a discrete number and unit of measurement
☒	☐	A statement on whether measurements were taken from distinct samples or whether the same sample was measured repeatedly
☐	☒	The statistical test(s) used AND whether they are one- or two-sided<br>*Only common tests should be described solely by name; describe more complex techniques in the Methods section.*
☐	☒	A description of all covariates tested
☐	☒	A description of any assumptions or corrections, such as tests of normality and adjustment for multiple comparisons
☐	☒	A full description of the statistical parameters including central tendency (e.g. means) or other basic estimates (e.g. regression coefficient) AND variation (e.g. standard deviation) or associated estimates of uncertainty (e.g. confidence intervals)
☒	☐	For null hypothesis testing, the test statistic (e.g. *F*, *t*, *r*) with confidence intervals, effect sizes, degrees of freedom and *P* value noted<br>*Give P values as exact values whenever suitable.*
☒	☐	For Bayesian analysis, information on the choice of priors and Markov chain Monte Carlo settings
☒	☐	For hierarchical and complex designs, identification of the appropriate level for tests and full reporting of outcomes
☒	☐	Estimates of effect sizes (e.g. Cohen's *d*, Pearson's *r*), indicating how they were calculated

Our web collection on statistics for biologists contains articles on many of the points above.

Software and code

Policy information about availability of computer code

Data collection	No software was used for the data collection
Data analysis	WGS data was aligned with BWA version 0.6.2, SAMtools version 0.1.18 and GATK 2.3.9. Retrotransposition calls with TraFiC 1.0, LINE-1 transductions with DELLY version 0.09, RNA data was processed with Kallisto version 0.43.0.

For manuscripts utilizing custom algorithms or software that are central to the research but not yet described in published literature, software must be made available to editors/reviewers. We strongly encourage code deposition in a community repository (e.g. GitHub). See the Nature Research guidelines for submitting code & software for further information.

Data

Policy information about availability of data

All manuscripts must include a data availability statement. This statement should provide the following information, where applicable:
- Accession codes, unique identifiers, or web links for publicly available datasets
- A list of figures that have associated raw data
- A description of any restrictions on data availability

The whole-genome point mutations have been deposited in the EGA database under the accession code EGAS00001003010 [https://ega-archive.org/studies/EGAS00001003010]. Gene expression values have been deposited in the Zenodo database under the Digital Object Identifier [10.5281/zenodo.3241399] [https://zenodo.org/deposit/3241399]. The methylation source data and the data underlying Fig3 are provided as Source Data files. All the other data supporting the findings of this study are available within the article and its Supplementary Information files. A reporting summary of this article is available as a Supplementary Information file.

Field-specific reporting

Please select the one below that is the best fit for your research. If you are not sure, read the appropriate sections before making your selection.

☒ Life sciences ☐ Behavioural & social sciences ☐ Ecological, evolutionary & environmental sciences

For a reference copy of the document with all sections, see nature.com/documents/nr-reporting-summary-flat.pdf

Life sciences study design

All studies must disclose on these points even when the disclosure is negative.

Sample size	Whole genome sequencing was performed in the maximum amount of samples possible under the funding constraint.
Data exclusions	Only samples with random missing data were excluded.
Replication	Our WGS CRC cohort is at present unique in size and a validation cohort is not available.
Randomization	Not applicable. No experiments were done in this study
Blinding	A list of 100 random insertions was given blindly to another researcher to evaluate the sensitivity of TraFiC calls

Reporting for specific materials, systems and methods

We require information from authors about some types of materials, experimental systems and methods used in many studies. Here, indicate whether each material, system or method listed is relevant to your study. If you are not sure if a list item applies to your research, read the appropriate section before selecting a response.

Materials & experimental systems

n/a	Involved in the study	
☒	☐	Antibodies
☒	☐	Eukaryotic cell lines
☒	☐	Palaeontology
☒	☐	Animals and other organisms
☐	☒	Human research participants
☒	☐	Clinical data

Methods

n/a	Involved in the study	
☒	☐	ChIP-seq
☒	☐	Flow cytometry
☒	☐	MRI-based neuroimaging

Human research participants

Policy information about studies involving human research participants

Population characteristics	All clinical data is collected in Supplementary Table 5.
Recruitment	Samples were obtained from a population based series and from a subsequently collected series of colorectal cancer cases.
Ethics oversight	All samples were collected after informed consent. In the great majority of cases the consent was signed, in few cases collected before 1999 a verbal consent was derived (signed informed consent was not required in the Finnish legislation prior to that). For these early samples subsequent authorization for research use was derived from the National Supervisory Authority for Welfare and Health (Dnro 421/04/044/06, Dnro 8048/06.01.03.01/2014, Dnro 358/32/300/05, Dnro 1476/06.01.03.01/2012). The study has been reviewed by the Ethics Committee of the Hospital district of Helsinki and Uusima (Dnro 133/E8/03, 408/13/03/03/2009). Permission to use patient information was obtained from the National Institute for Health and Welfare (Dnro 53/07/2000, Dnro THL/1071/5.05.00/2011, Dnro THL/151/5.05.00/2017).

Note that full information on the approval of the study protocol must also be provided in the manuscript.
